# *Stellera chamaejasme* L. extract induces apoptosis of human lung cancer cells via activation of the death receptor-dependent pathway

**DOI:** 10.3892/etm.2012.643

**Published:** 2012-07-20

**Authors:** XIAONI LIU, XIAOXIN ZHU

**Affiliations:** 1 Beijing Institute of Liver Diseases and; 2 Beijing YouAn Hospital, Capital Medical University, Beijing 100069;; 3 Institute of Chinese Materia Medica, China Academy of Chinese Medical Sciences, Beijing 100700, P.R. China

**Keywords:** *Stellera chamaejasme* L., apoptosis, tumor, death receptor, natural product

## Abstract

*Stellera chamaejasme* L. has been widely used in the treatment of lung, liver and esophageal cancer in Chinese traditional medicine. In our previous study, we found that the extract of *Stellera chamaejasme L*. (ESC) inhibited the growth and induced the apoptosis of human lung cancer NCI-H157 cells. In the present study, we investigated the cellular effects of an ESC-2 extract isolated from ESC in the NCI-H157 human lung cancer cell line. We found that ESC-2 inhibited the growth of NCI-H157 cells, demonstrating ESC-2-induced morphological changes in cells and reduced cell viability in a dose- and time-dependent manner. Furthermore, we observed that ESC-2 resulted in apoptosis, activation of caspase-8 and caspase-3, and an increase in Fas expression, indicating that the NCI-H157 cell growth inhibitory activity of ESC-2 was due to death receptor-dependent pathway-mediated apoptosis, which may partly explain the anti-cancer activity of ESC-2.

## Introduction

It is the goal of natural medicine researchers to trace active ingredients from natural products and then develop clinically effective drugs. The earliest drugs in human use have been obtained from natural products. From ancient times to the present, natural medicines have been developed and even now play an extremely important role in daily medication (1–3). It is reported that 25% of prescription drugs in the US contain active ingredients derived from plants. In 1973, 13.3% and 2.7% were derived from microorganisms and animals, respectively. Half of the best-selling drugs in 1991 were from natural compounds or derivatives. To date, only 5–15% of the 250,000 types of higher plants have been screened (4). As such, natural medicine has great potential and much room for development.

Natural products have varied forms of biological activity. Anti-tumor activity is a very important research subject. Cancer treatment is a topical and complex area of modern study and chemotherapy remains a commonly used method for the treatment of cancer. More than 60% of cancer chemotherapeutic agents currently in use are natural products or derivatives (5) such as vincristine, paclitaxel and camptothecin. These anti-cancer drugs from natural products have been used clinically and the effects are quite significant (6).

Cell death by apoptosis eliminates excess, redundant or abnormal cells in animals and hence is crucial for animal development and tissue homeostasis. Disturbed regulation of this vital process represents a major causative factor in the pathogenesis of cancers (7). There are two apoptotic pathways: the death receptor pathway and the mitochondrial pathway. Death receptors are cell surface receptors that belong to the tumor necrosis factor (TNF) superfamily, which trigger apoptosis upon ligand binding. The best characterized death receptors are Fas (CD95/Apo1), TNF receptor 1 (p55), TRAMP (WSL-1/Apo3/DR3/LARD), TRAIL-R1 (DR4) and TRAIL-R2 (DR5/Apo2/KILLER). Death receptors contain an intracellular death domain (DD), which upon ligand binding (Fas ligand, TNF, TWEAK, TRAIL) associates with an adaptor protein called Fas-associated death domain (FADD) directly or indirectly via the TNFR-associated death domain (TRADD). FADD also interacts with pro-caspase-8 to form the death inducing signaling complex (DISC) at the receptor. Once assembled, DISC induces the activation of caspase-8, which in turn precipitates the activation of downstream effector caspases (8–12). Therefore, promoting cell apoptosis via regulation of the TNF death receptor family proteins has been the main focus in the development of anti-cancer therapies.

Traditional Chinese medicine is an important part of the field of natural medicine and has a clear application in anti-tumor treatment. *Stellera chamaejasme* L. known as ‘Rui Xiang Lang Du’, a pungent Chinese herb, with the effects of detoxification, edema alleviation, elimination of slough and promotion of granulation, has long been used for the treatment of various tumors in the Chinese population. In a previously published study, we found that ethanol extract of *Stellera chamaejasme* L. (ESC) inhibited the growth of H_22_ and BEL-7402 tumor cells and induced apoptosis of BEL-7402 tumor cells *in vitro* and *in vivo* (13).

In this study, we obtained ESC-2 from ESC using a special isolation technique. In order to further trace and confirm the potential anti-cancer biological activity of *Stellera chamaejasme* L., we evaluated the inhibitory effect of ESC-2 on the growth of NCI-H157 human lung cancer cells and investigated the possible apoptotic molecular mechanisms mediating its biological effect *in vitro*.

## Materials and methods

### Materials and reagents

RPMI-1640 medium, fetal bovine serum (FBS), penicillin-streptomycin and Trypsin-EDTA were purchased from Gibco (Grand Island, NY, USA). Sulforhodamine B, propidium iodide (PI) and RNase were provided by Sigma Chemical Co. (St. Louis, MO, USA). A fluorescein isothiocyanate (FITC)-conjugated Annexin V apoptosis detection kit was provided by Becton-Dickinson (San Jose, CA, USA). Caspase-3, -8 colorimetric protease assay kits were purchased from Chemicon International, Inc. (Temecula, CA, USA). Fas, TRAIL-R1 ELISA kit were from R&D Systems (Minneapolis, MN, USA). All the other chemicals used, unless otherwise stated, were obtained from Sigma Chemicals.

### Preparation of the extract from Stellera chamaejasme L. Stellera chamaejasme

L. herbal medicine was extracted three times with 100% ethanol and the resulting liquid (volatiled to ensure a non-alcohol status) was washed in a polyamide column with 60% ethanol, which were recycled decompressively and vacuum dried at room temperature. The compound finally obtained was ESC (13). ESC was mixed with reverse silica, filtered through a C18 column and eluted with 100% methanol; the final samples being ESC-2.

### Cell culture

Human lung cancer NCI-H157 cells were obtained from the Cell Culture Center of the Chinese Academy of Medical Sciences. The cells were grown in RPMI-1640 medium containing 10% (v/v) FBS and 100 U/ml penicillin and 100 *μ*g/ml streptomycin in a 37°C humidified incubator with 5% CO_2_. The cells were subcultured at 80–90% confluency. Cells used in this study were subjected to no more than 20 cell passages.

### Evaluation of cell viability by the sulforhodamine B assay

Cell viability was assessed by the sulforhodamine B (SRB) colorimetric assay. NCI-H157 cells were seeded into 96-well plates at a density of 0.5×10^4^ cells/well in 0.1 ml medium. The cells were treated with various concentrations of ESC-2 for different time periods. Treatment with 0.2% DMSO was included as a vehicle control. Following addition of ESC-2, the plates were incubated for an additional 48 h. The assay was terminated by the addition of cold trichloroacetic acid (TCA). Cells were fixed *in situ* by the addition of 50 *μ*l of cold 50% (w/v) TCA (final concentration, 10%) and incubated for 1 h at 4°C. Then 100 *μ*l SRB solution at 0.4% (w/v) in 1% acetic acid was added to each well, and the plates were incubated for 10 min at room temperature. Following staining, unbound dye was removed by washing five times with 1% acetic acid and the plates were air dried. Bound stain was subsequently solubilized with 10 mM non-buffered tris-acetate (pH 10.5) and the absorbance was read on an automated plate reader (ELX800 type, Bio-Tex Instruments, Inc.) at a wavelength of 515 nm. The inhibition rate (%) was calculated as follows:

Inhibition rate (%)=1−mean absorbance of treatment group/mean absorbance of vehicle control group.

### Observation of morphological changes

NCI-H157 cells were seeded into 96-well plates at a density of 0.5×10^5^ cells/well in 0.1 ml medium. The cells were treated with various concentrations of ESC-2 for 48 h. Cell morphology was observed using an inverted microscope (Olympus, Japan). The images were captured at a magnification of x200.

### Detection of apoptosis by flow cytometric analysis with PI staining

Following incubation with various concentrations of ESC-2, 1×10^5^/ml cells were collected into a single-cell suspension. Pre-cooled 70% ethanol was added to fix cells at 4°C for 24 h or more. Cells were rinsed 2 times with 500 *μ*l PBS prior to being stained with a solution containing 50 *μ*g/ml PI and 100 *μ*g/ml RNase for 30 min in the dark, and subsequently apoptosis of the NCI-H157 cells was determined by flow cytometric analysis using a fluorescence-activated cell sorting (FACS) caliber (Becton-Dickinson, USA).

### Observation of apoptosis by fluorescence microscopy with Annexin V/PI staining

Following incubation with various concentrations of ESC-2, apoptosis of NCI-H157 cells was observed by fluorescence microscopy and Annexin V-fluorescein isothiocyanate (FITC)/PI kit. Staining was performed according to the manufacturer’s instructions. The early apoptotic cells were labeled by green fluorescence (FITC-labeled), while the late apoptotic cells were labeled by both green and red fluorescence (the latter PI-labeled).

### Analysis of caspase activation

The activities of caspase-3 and -8 were determined by a colorimetric assay using the caspase-3 and -8 activation kits, following the manufacturer’s instructions. Briefly, following treatment with various concentrations of ESC-2 for 24 h, NCI-H157 cells were lysed with provided lysate buffer for 30 min on ice. The lysed cells were centrifuged at 16,000 × g for 10 min, and 100 *μ*g of the protein was incubated with 50 *μ*l of the colorimetric tetrapeptides, Asp-Glu-Val-Asp (DEAD)-p-nitroaniline (pNA) (specific substrate of caspase-3) or Ile-Glu-Thr-Asp (IETD)-pNA (specific substrate of caspase-8) at 37°C in the dark for 2 h. Samples were read at 405 nm in an ELISA reader (BioTek, Model EXL800, USA). The data were normalized to the activity of the caspases in the control cells (treated with 0.5% DMSO vehicle) and presented as the fold of the control.

### ELISA analysis

The level of Fas and TRAIL-R1 in the cells was determined using an enzyme-linked immunosorbent assay (ELISA) kit according to the manufacturer’s protocol. Briefly, the cell supernatant together with 9-times sample buffer (100 *μ*l) were added to the corresponding sample well of the microtiter plate. Plates were incubated at 37°C for 2 h. When the antigens present in the sample had bound to the coating antibodies adsorbed in the microwells, the plates were washed with 100 *μ*l of wash buffer five times. Microtiter plates to which were added 100 *μ*l biotinylated antibody were incubated at 37°C for 1 h. Plates were washed again. Conjugated enzymes (100 *μ*l) were added to bind to antigens captured by the biotinylated antibody. Following the removal of unbound conjugated enzyme antibody by the same washing steps, 100 *μ*l TMB solution was added. Following incubation at room temperature in the dark for 15 min, the reaction was stopped by adding 100 *μ*l stop solution, and the absorbance was measured at 450 nm with a microplate reader. The concentrations of Fas and TRAIL-R1 in the samples were determined on the base of a standard curve prepared from seven standard dilutions. All samples were analyzed in duplicate, and the limit of detection was determined to be 15 pg/ml.

### Statistical analysis

All data are the means of three determinations and data were analyzed using the SPSS Package for Windows (Version 12). Statistical analysis of the data was performed with ANOVA. Differences with P<0.05 were considered statistically significant.

## Results

### ESC-2 inhibits the growth of NCI-H157 cells

**Figure 1:**
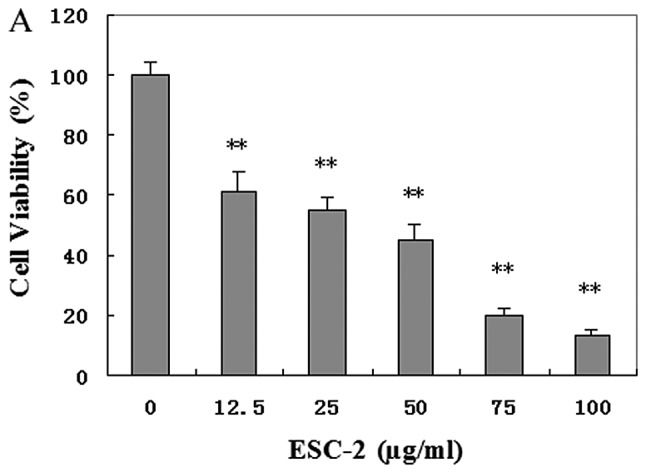

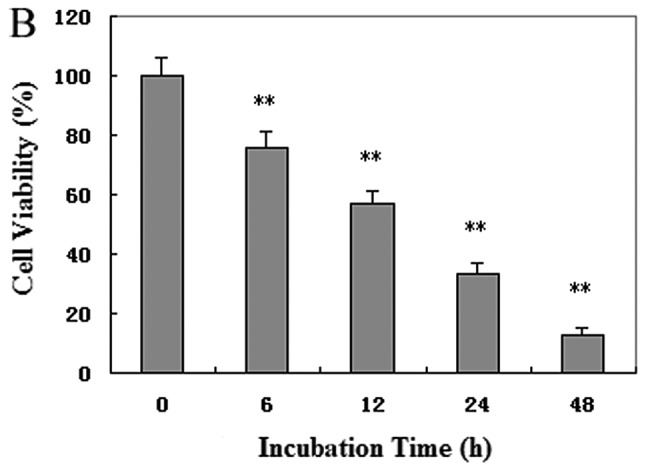
Effect of ESC-2 on the viability of NCI-H157 cells. (A) NCI-H157 cells were treated with the indicated concentrations of ESC-2 for 48 h. (B) Cells were treated with 50 *μ*g/ml of ESC-2 for the indicated time periods. Cell viability was determined by the SRB assay. The data were normalized to the viability of control cells (100%, treated with 0.2% DMSO vehicle). Data are averages with SD (error bars) from at least three independent experiments. ^*^P<0.05, ^**^P<0.01, significant versus control cells.

**Figure 2:**
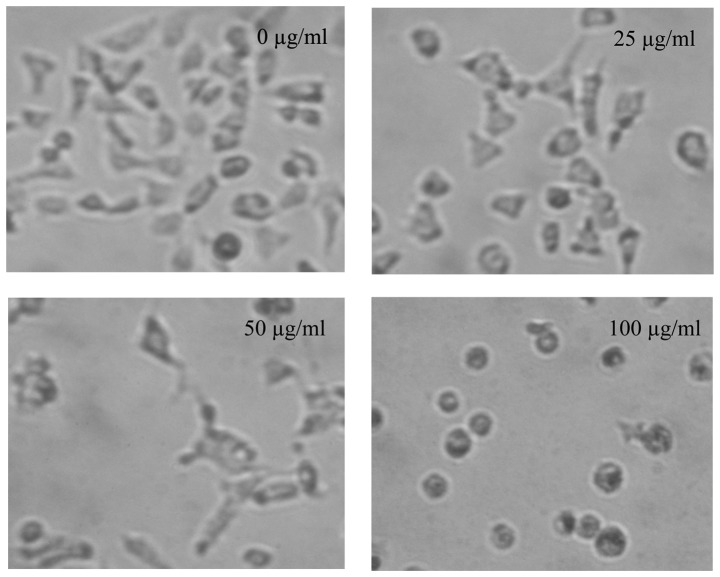
Effect of ESC-2 on the morphological changes of NCI-H157 cells. NCI-H157 cells were treated with the indicated concentrations of ESC-2 for 24 h and morphological changes were observed using inverted microscopy. The images were captured at a magnification of x200. Images are representative of three independent experiments.

The effect of ESC-2 on the viability of NCI-H157 cells was determined by SRB assay. As shown in Fig. 1A, treatment with 12.5–100 *μ*g/ml of ESC-2 for 48 h dose-dependently reduced cell viability by 13.26–61.49% compared to the untreated control cells (P<0.01), with an estimated half-maximal inhibitory concentration (IC_50_) value of 50 *μ*g/ml. The cell viability was decreased to 61.49% at the highest concentration of ESC-2 (100 *μ*g/ml) in this study. We also evaluated the effect of 50 *μ*g/ml of ESC-2 (IC_50_ value) on cell viability with incubation for different periods of time. As shown in Fig. 1B, treatment with 100 *μ*g/ml of ESC-2 led to a gradual decrease in cell viability with the increase of exposure time. These results suggest that ESC-2 inhibits NCI-H157 cell growth or viability in a dose-and time-dependent manner. To further verify these results, we evaluated the effect of ESC-2 on NCI-H157 cell morphology via inverted microscopy, since cell morphology in culture is indicative of the healthy status of the cells. As shown in Fig. 2, untreated NCI-H157 cells appeared as densely packed and disorganized multilayers, whereas following incubation with various concentrations of ESC-2 for 24 h a number of the cells became rounded and shrunken, and detached from each other or floated in the medium. In addition, after a 24 h exposure to ESC-2, the cells became less confluent. Taken together, these data demonstrate that ESC-2 inhibits the growth of NCI-H157 cells.

### ESC-2 induces apoptosis of NCI-H157 cells

**Figure 3:**
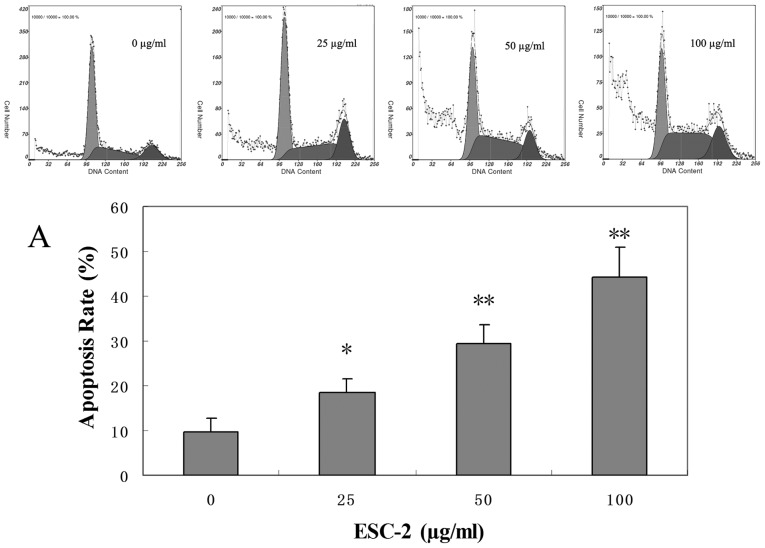

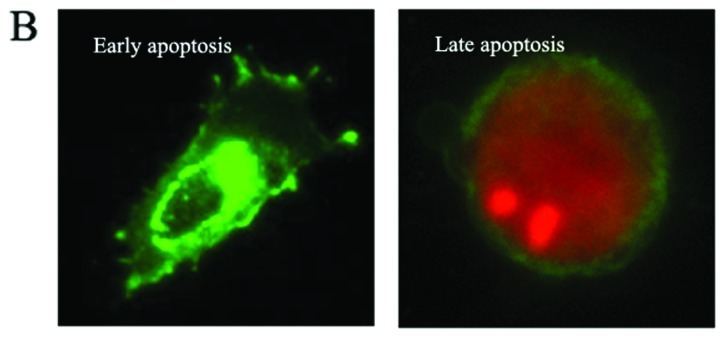
Apoptosis induced by ESC-2 in NCI-H157 cells. (A) NCI-H157 cells were treated with the indicated concentrations of ESC-2 for 24 h and apoptosis rates were observed using flow cytometry. Data are averages with SD (error bars) from at least three independent experiments. ^*^P<0.05, ^**^P<0.01, significant versus control cells. (B) Cells were treated with 50 *μ*g/ml of ESC-2 for 24 h and apoptotic phenomena were observed with fluorescence microscope. Early apoptotic cells were labeled by green fluorescence (FITC-labeled), while the late apoptotic cells were labeled by both green and red fluorescence (the latter PI-labeled). The images were captured at a magnification of ×200.

To determine whether the cell-growth suppressive effect of ESC-2 is due to apoptosis, we examined the pro-apoptotic activity of ESC-2 in NCI-H157 cells via PI staining followed by FACS analysis. As shown in Fig. 3A, the percent of cells undergoing apoptosis following treatment with 0, 25, 50 and 100 *μ*g/ml of ESC-2 for 24 h was 5.68, 18.57, 29.54 and 44.13%, respectively (P<0.05, versus untreated control cells). This indicates that ESC-2 treatment induces NCI-H157 cell apoptosis in a dose-dependent manner. To confirm the pro-apoptotic function of ESC-2, we further investigated the pro-apoptotic effect of ESC-2 with Annexin/PI double staining. As shown in Fig. 3B, NCI-H157 cells treated with 50 *μ*g/ml of ESC-2 (IC_50_ value) for 24 h displayed the characteristic green and red fluorescent staining of early and late apoptosis.

### ESC-2 induces activation of caspase-8 and -3

**Figure 4:**
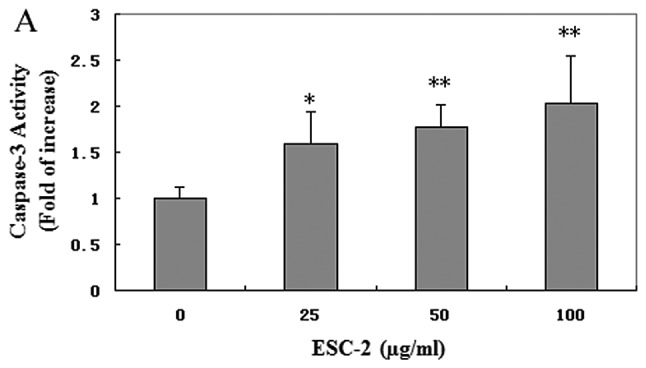

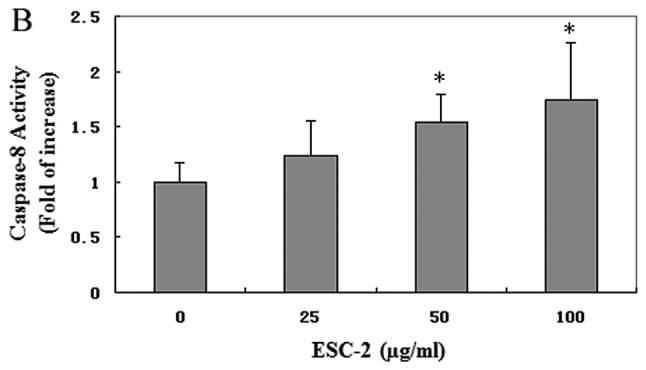
Effect of ESC-2 on the activity of caspases in NCI-H157 cells. The cells were treated with the indicated concentrations of ESC-2 for 24 h. Caspase-3 and -8 activities were determined by a colorimetric assay. The data were normalized to the caspase activities within control cells (treated with 0.2% DMSO vehicle) and represented as fold of control. Data are averages with SD (error bars) from at least three independent experiments. ^*^P<0.05, ^**^P<0.01, significant versus control cells.

To identify the downstream effectors in the apoptotic signaling pathway, the activation of caspase-8 and -3 was examined by a colorimetric assay using specific chromophores, DEVD-pNA (specific substrate of caspase-3) and IETD-pNA (specific substrate of caspase-8). As shown in Fig. 4A and B, ESC-2 treatment significantly and dose-dependently induced activation of both caspase-8 and -3 in the NCI-H157 cells (P<0.01 or 0.05, versus untreated control cells). These data suggest that ESC-2 promotes NCI-H157 cell apoptosis via the death receptor-dependent pathway.

### ESC-2 regulates the expression of Fas and TRAIL-R

**Figure 5:**
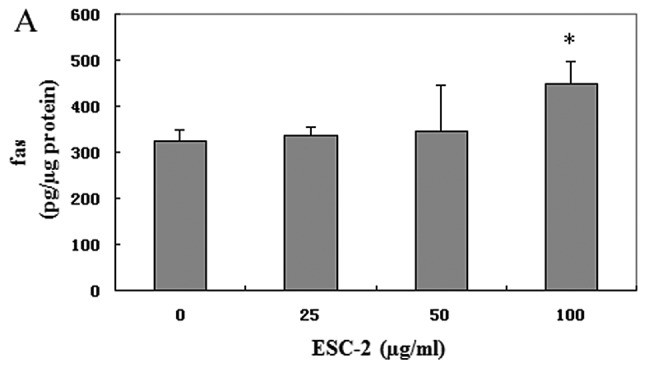

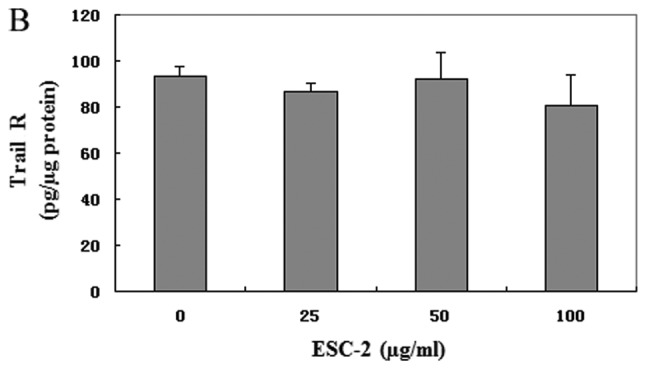
Effect of ESC-2 on the death receptor protein expression in NCI-H157 cells. The cells were treated with the indicated concentrations of ESC-2 for 24 h. Fas and Trail-R expression was determined by ELISA assay. Data are averages with SD (error bars) from at least three independent experiments. ^*^P<0.05, ^**^P<0.01, significant versus control cells.

To further study the mechanism of the anti-cancer activity of ESC-2, we examined the protein expression level of death receptor of Fas and TRAIL-R1 in ESC-treated NCI-H157 cells using the ELISA assay. The results showed that ESC-2 treatment profoundly increased Fas expression (Fig. 5A), while it did not obviously influence TRAIL-R1 expression in NCI-H157 cells (Fig. 5B).

## Discussion

In recent years, traditional Chinese herbal remedies have gradually gained considerable attention as a new source of anti-cancer drugs. Although their healing mechanisms are still largely unknown, some of the drugs have been used advantageously to help cancer patients fight their disease when compared to other treatments (14). *Stellera chamaejasme* L. is one of these herbs, widely used in the Chinese population to treat various types of cancer and other diseases. Our previously published study confirmed that extracts of *Stellera chamaejasme* L. (ESCs) have significant anti-tumor effects *in vitro* and *in vivo* (13,15). Here, we reported that ESC-2 (extracted from ESC) reduces the viability and inhibits growth of NCI-H157 cells in a dose-and time-dependent fashion and may be a potential anti-tumor drug candidate. Therefore, since ESC-2 is able to be further developed as an anti-cancer agent, its underlying molecular mechanism should be elucidated. Cytotoxic drugs often used in chemotherapy of tumors cause apoptosis in target cells. In this study, we demonstrated that these anti-cancer effects on NCI-H157 cells resulted from the induction of apoptosis by ESC-2.

We used Annexin V/PI double staining to observe the apoptotic phenomena and PI single staining to assess the apoptosis rate in NCI-H157 cells. Our data clearly showed that treatment with ESC-2 leads to apoptosis in a dose-dependent manner.

Caspases, represented by a family of cysteine proteases, are the key proteins that modulate the apoptotic response. Caspase-3 is a key executioner of apoptosis, which is activated by an initiator caspase such as caspase-8 during death receptor-mediated apoptosis (16). In the present study, we found that ESC-2 induces the activation of both caspase-8 and -3 in NCI-H157 cells in a dose-dependent manner. Thus, ESC-2-induced NCI-H157 cell death is accompanied by an increase in the activities of caspase-8 and -3, which then stimulates the molecular cascade for apoptosis.

Death receptor-dependent apoptosis is mainly regulated by tumor necrosis factor superfamily proteins. In this study, we detected the two classical death receptor, Fas and TRAIL-R1, in NCI-H157 cells.

The Fas (APO-1/CD95)/Fas ligand (CD95-L) system is a key regulator of apoptosis (17). Fas is also known as CD95, Apo-1, and tumor necrosis factor receptor superfamily, member 6 (TNFRSf6). Fas is located on chromosome 10 in humans and 19 in mice. Similar sequences related by evolution (orthologs) are found in most mammals (18). Studies have shown that increased expression of fas protein on the tumor cell surface can promote apoptosis of tumor cells (19). Here we describe that ESC-2 induces apoptosis via upregulation of Fas expression in the human NCI-H157 cell line. The finding that apoptosis caused by ESC-2 may be mediated via the Fas system provides a molecular insight into the research of the mechanisms of ESC-2 in tumor treatment. TRAIL-R is another death receptor recently identified that also belongs to the tumor necrosis factor receptor superfamily. It activates caspase-8 to induce apoptosis by binding its ligand TRAIL (20). To date, five types of TRAIL receptors have been identified: TRAIL-R1, TRAIL-R2, TRAIL-R3, TRAIL-R4 and OPG. TRAIL-R1 and TRAIL-R2 have a death domain and are able to transfer apoptotic signals and subsequently induce apoptosis (21,22). In the present study, we evaluated TRAIL-R1 expression. The results showed that ESC-2 did not obviously influence TRAIL-R1 expression. We suggest that the regulating mechanism of ESC-2 in apoptosis is complex, and the pro-apoptotic effect of ESC-2 may be the result of the integrated regulation of the tumor apoptosis pathway protein expression.
